# Proteomic and Bioinformatic Studies for the Characterization of Response to Pemetrexed in Platinum Drug Resistant Ovarian Cancer

**DOI:** 10.3389/fphar.2018.00454

**Published:** 2018-05-08

**Authors:** Leda Severi, Lorena Losi, Sergio Fonda, Laura Taddia, Gaia Gozzi, Gaetano Marverti, Fulvio Magni, Clizia Chinello, Martina Stella, Jalid Sheouli, Elena I. Braicu, Filippo Genovese, Angela Lauriola, Chiara Marraccini, Alessandra Gualandi, Domenico D'Arca, Stefania Ferrari, Maria P. Costi

**Affiliations:** ^1^Department of Life Sciences, University of Modena and Reggio Emilia, Modena, Italy; ^2^Department of Biomedical Science, Metabolic Science and Neuroscience, University of Modena and Reggio Emilia, Modena, Italy; ^3^Department of Medicine and Surgery, University of Milano Bicocca, Monza, Italy; ^4^Department of Gynecology, European Competence Center for Ovarian Cancer, Charité—Universitätsmedizin Berlin, Berlin, Germany; ^5^Centro Interdipartimentale Grandi Strumenti, University of Modena and Reggio Emilia, Modena, Italy

**Keywords:** pemetrexed, folate pathway, drug resistance, ovarian cancer, proteomics, mass spectrometry, bioinformatics

## Abstract

Proteomics and bioinformatics are a useful combined technology for the characterization of protein expression level and modulation associated with the response to a drug and with its mechanism of action. The folate pathway represents an important target in the anticancer drugs therapy. In the present study, a discovery proteomics approach was applied to tissue samples collected from ovarian cancer patients who relapsed after the first-line carboplatin-based chemotherapy and were treated with pemetrexed (PMX), a known folate pathway targeting drug. The aim of the work is to identify the proteomic profile that can be associated to the response to the PMX treatment in pre-treatement tissue. Statistical metrics of the experimental Mass Spectrometry (MS) data were combined with a knowledge-based approach that included bioinformatics and a literature review through ProteinQuest™ tool, to design a protein set of reference (PSR). The PSR provides feedback for the consistency of MS proteomic data because it includes known validated proteins. A panel of 24 proteins with levels that were significantly different in pre-treatment samples of patients who responded to the therapy vs. the non-responder ones, was identified. The differences of the identified proteins were explained for the patients with different outcomes and the known PMX targets were further validated. The protein panel herein identified is ready for further validation in retrospective clinical trials using a targeted proteomic approach. This study may have a general relevant impact on biomarker application for cancer patients therapy selection.

## Introduction

The folate pathway is an important biochemical target for anti-cancer drugs and overall more than 1,500 clinical trials are currently running (www.clinicaltrials.gov); therefore it represents an active wide field of study today (Wilson et al., 2014; Taddia et al., 2015).

After first line treatment of ovarian cancer (OC) patients with platinum drugs, drug resistance occurs rapidly inducing the overexpression of folate-dependent proteins such as thymidylate synthase (TS, TYSY) (Ozasa et al., 2010), dihydrofolate reductase (DHFR, DYR), the trifunctional purine biosynthetic protein adenosine-3 (GART, PUR2) and other enzymes involved in cell replication. Drugs directed against TYSY and the folate metabolism are being investigated as potential second-/third-line therapies for platinum drug-resistant OC. Among them pemetrexed (PMX, Alimta™) has been proposed with the rationale that it can target the mentioned proteins, thus blocking cancer cell replication and death.

PMX is already in use in colon and pancreatic cancer, as well as in mesothelioma patients sensitive to TS targeting drugs (https://www.drugbank.ca/), and it was proposed for use in a clinical phase II trial for the treatment of resistant OC (Vergote et al., 2009). A recent review (Egloff and Jatoi, 2014) examined patients with OC who were treated with PMX in a non-clinical trial setting in Germany; PMX demonstrated to improve the progression free-survival in a variable manner. The proper selection of the sub-population that could respond to the proposed therapy could improve the outcome, if suitable biomarkers to predict PMX response can be identified.

In our previous studies, using a combination of bioinformatics and MS proteomics we were able to show, in ovarian cancer cells sensitive and resistant to cis-platinum, that a new class of peptidic TS inhibitors induced a proteomic modulation pattern, when targeting TS, different with respect to other classical inhibitors, since they behave as protein-protein interaction inhibitors (Genovese et al., 2014). We identified a protein set that is specifically modulated when peptides are delivered to the cancer cells. The results of our study on the cancer cell models suggest that proteomics and bioinformatics are a useful combined technology for the characterization of TS inhibitors induced protein modulation and that this modulation is associated with their mechanism of action. This suggests the potential role of the proteomics and bioinformatics technologies applied to the study of the effect induced by a TS inhibitor on cancer cells. Therefore, the same technology can be applied to study human cancer tissues to study anti-folate drugs behavior in human tissue.

**Figure 1:**
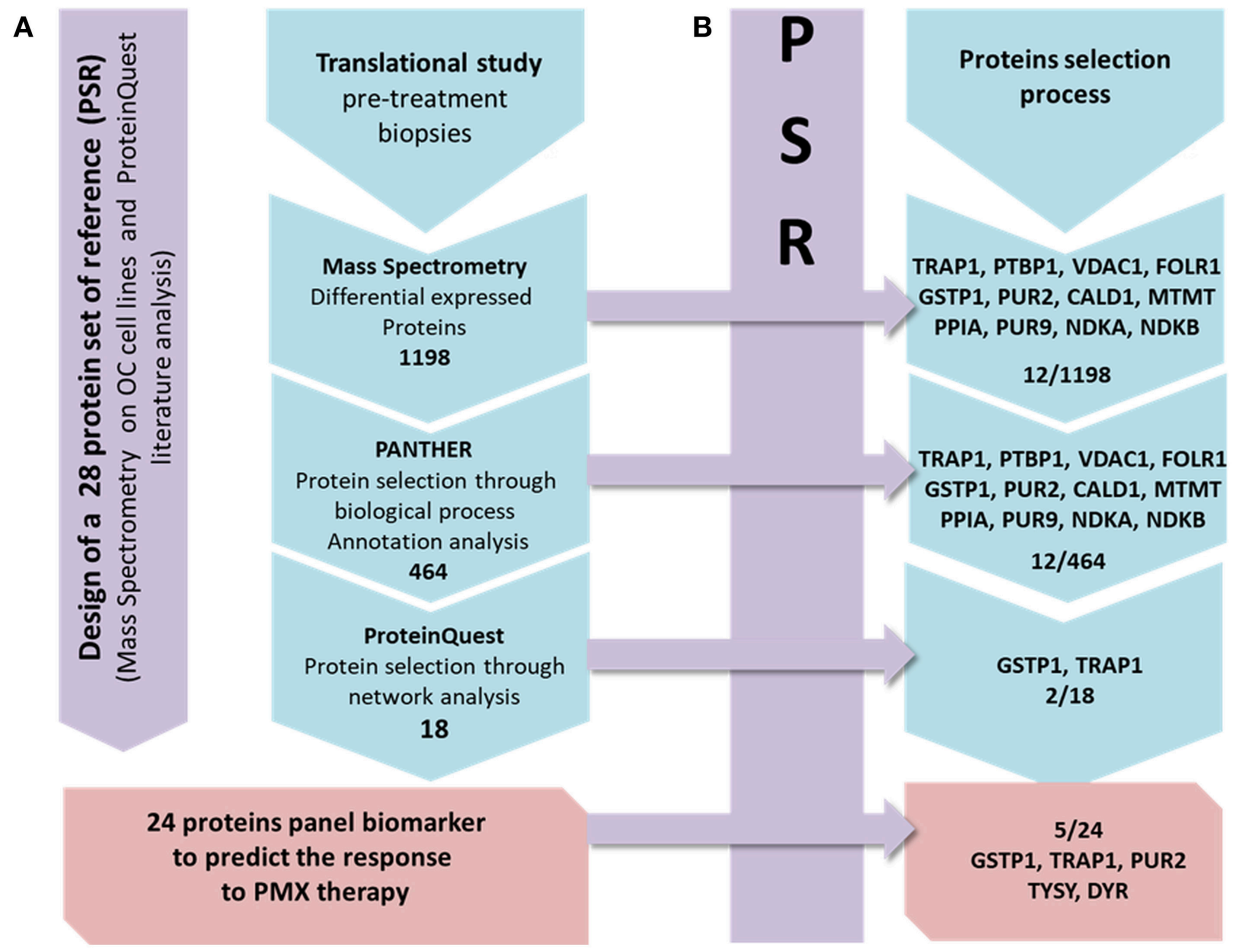
Workflow describing the strategy applied in the present study. From left to right: the first panel (violet) presents the design of the PSR, including 28 proteins. Line **(A)**: the MS protein selection process that combines the experimental and bioinformatics steps is presented in 4 frames. The action/software used to select the DEPs set obtained from the MS data to the final panel is shown. Line **(B)**: each frame contains the proteins of the PSR included in each frame of the process in line A.

In the present study we applied a previously developed approach (Genovese et al., 2014) to analyze tissue samples taken from patients affected by ovarian cancer, after failure of platinum drug and before second line therapy with PMX. We wanted to explore if a specific protein expression pattern exists in pre-treatment samples from patients who responded to the PMX therapy with respect to the ones who did not respond, and if the specific pattern can be explained in relation to the thymidylate synthase cellular role. We applied a label free MS proteomic analysis combined with bioinformatics (Figure 1A) and adopted the concept of protein set of references (PSR) to drive the selection and panel identification process (Figure 1B).

## Materials and methods

### Experimental design and statistical rationale

The proteomic study is based on tissue samples taken from retrospective study aimed to explore and identify a protein panel that is associated with the response to PMX therapy based on the analysis of pre-treatment ovarian biopsies. OC biopsies are collected from women with high-grade serous ovarian carcinoma, who were subjected, after surgery reduction, to homogenous first line chemotherapy consisting in carboplatin and paclitaxel, to variable second/third lines chemotherapy with or including PMX. During the collaboration within the EUTROC initiative we have received a total of 52 tissue samples from the TOC bank (https://forschungsberichte.charite.de/FOB_2010-2011/english/PJ/PJ49331.html; for the institutional Ethics Committee approval reference to TOC). We applied the sample selection protocol reported below and we could only select three samples that were further analyzed. A proteomic study was implemented with a two steps experiment. The first step was the design of a PSR that is compiled through literature-based analysis focusing on PMX experimentally detected proteins involved in the mechanism of action or relevant marker of its activity (Figure 1A). It was additionally enriched with experimental proteins modulated in OC cell lines when treated with PMX performed during a previous work (Taddia, 2015). Identification of a protein subset that characterizes the activity of folate metabolism targeted Ph.D. thesis. University of Modena and Reggio Emilia (unpublished data). The second step was the label-free MS proteomic study of the tissues in which a stepwise protein selection was performed following the criteria presented below (Figure 1B). Monitoring of the stepwise selection consistency is performed by comparison with the PSR. The final panel was then obtained and three proteins were further validated using a western-blot analysis.

### Sample selection

All the 52 tissue samples were histologically analyzed. Only non-fibrous OC tissue samples that showed a percentage of cancer cells >80% were further considered. Uniformity of the cancer cells percentage among biopsies is necessary to perform a differential analysis of the whole proteomes. We decided to consider only biopsies from patients who were subjected, before surgery reduction, to homogenous first line chemotherapy consisting in carboplatin and paclitaxel and, eventually, to a second line chemotherapy, and after surgery reduction to a variable chemotherapy with or including PMX. The clinical response to PMX chemotherapy was evaluated using RECIST (Response Evaluation Criteria In Solid Tumors) criteria after 6 months from the end of the treatment by imaging methods and the patients were divided into responder (CR, complete or PR, partial responder) and not-responder (NR, with progressive disease) according to RECIST. For proteomic studies, we considered only biopsies taken before PMX treatment as those taken after the treatment were collected between 1 and 24 months after the end of the treatment; thus, performing a post/pre differential proteomic analysis between samples from the same patient was not feasible due to high differences in sample collection.

### Tissue samples preparation

Fifty-two frozen biopsies of ovarian carcinoma stored at −80°C belonging to 24 patients were analyzed in order to confirm the diagnosis of high-grade serous ovarian carcinoma and to define the percentage of cancer cells in every sample. The biological specimens consisted of cubes approximately 0.5 cm on each side, with a weight of around 0.5 g. Each biopsy was cut in halves using a scalpel. Half was immediately put in dry ice and stored at −80°C for the MS analysis. The other piece was immersed in octanol and was sectioned at 4 μm in a cryostat. Haematoxylin/Eosin (HE) staining was performed on slices obtained and evaluated by the pathologist (LL). Samples and clinical data information were obtained from TOC.

For MS analyses, 20 μg of biopsy was chopped through a scalpel and then quickly weighted to keep it frozen. The piece was placed in a 2 mL microcentrifuge tube containing 1 stainless steel bead (5 mm mean diameter) previously kept on dry ice for at least 15 min. The tube was placed into a TissueLyser LT (Qiagen) adapter, and incubated at room temperature for 2 min. 600 μL of RIPA lysis buffer [20 mM TRIS-HCl, pH 7.5; 150 mM NaCl; 1 mM Na_2_EDTA (Ethylenediaminetetraacetic Acid);1 mM EGTA (Ethylene glycol-bis(β-aminoethyl ether)-N,N,N′,N′-tetra acetic acid tetrasodium salt]; 1% NP-40 (Tergitol™, Sigma); 1% sodium deoxycholate; 1 mM Na3VO4; 1 mM PMSF (Phenylmethylsulfonyl fluoride); Protease inhibitor cocktail (Sigma); Phosphatase Inhibitor Cocktail 2 and 3 (Sigma Aldrich) were added, then the tissues were homogenated through the use of TissueLyser LT (Quiagen) operating at 30 Hz for 1 × 2 min. Next, the homogenate was centrifuged at 16,000 r.c.f. for 20 min in a precooled microcentrifuge at 4°C, the supernatant was collected and the debris were discarded. Proteins extracted were quantified using a Bradford assay (Sigma Aldrich).

### Protein digestion via filter-aided sample preparation (FASP)

Protein digestion was performed following the protocol reported in literature (Wiśniewski et al., 2009). 200 μg of a protein extract of each sample and 200 μl of 8 M urea in 0.1 M Tris/HCl pH 8.5. (UA) containing DTT (1,4-Dithiothreitol, Sigma Aldrich) 0.1 M were mixed, and incubated for 30 min at 56°C to reduce thiol groups. The solution was loaded on a Microcon YM-30 ultrafilter (Millipore), and centrifuged at 14,000 r.c.f. for 15 min, and centrifugation was repeated after adding 200 μl of UA to the filter unit. For the alkylation, 100 μl of a 0.05 M iodoacetamide (IAA, Sigma Aldrich) solution in UA were added and incubated without mixing for 20 min in the dark. Filter units were centrifuged at 14,000 r.c.f for 10 min, washed twice adding 100 μl of UA to the filter unit, and centrifuged again at 14,000 r.c.f for 15 min.

To raise the pH, 100 μl of ABC (Ammonium BiCarbonate) 50 mM (Sigma Aldrich) were added to the filter unit and centrifuged at 14,000 r.c.f. for 10 min. This step was repeated twice. For the digestion, 40 μl of ABC with trypsin (Promega) were added (enzyme to protein ratio 1:50 w/w) and mixed at 600 r.p.m. in a thermo-mixer for 1 min. The filter units were incubated in a wet chamber at 37°C overnight, then they were transferred to new collection tubes, and centrifuged at 14,000 r.c.f. for 40 min. After adding 40 μl ABC, the filter units were centrifuged again at 14,000 r.c.f. for 10 min. Finally, the pooled extracts were acidified with trifluoroacetic acid and desalted using solid phase extraction (SPE) cartridges (C18-SD 7 mm/3 mL). The peptides were lyophilized using Eppendorf Concentrator plus (Eppendorf). The samples were subsequently suspended in 75 μL of an appropriate mobile phase (98% H_2_O:2% acetonitrile: 0.1% trifluoroacetic acid) and analyzed on a Ultra-High Resolution Qq-Time-Of-Flight mass spectrometer (UHR-QqTOF).

### LC MS/MS label-free quantification

Desalted peptide mixtures samples were suspended and quantificated by NanoDrop assay. Each sample (1 μg) was analyzed at least three times into a Dionex nRSLC (Rapid Separation LC nano, Thermo Scientific, Sunnyvale, CA), coupled online with an Impact HDTM UHR-QqToF (Bruker Daltonics, Germany). Digests were loaded onto a pre-column (Dionex, Acclaim PepMap 100 C18, cartridge, 300 μm) and then separated using a 50 cm column (Dionex, ID 0.075 mm, Acclaim PepMap100, C18) at 40°C using a 360 min multistep gradient at a flow rate of 300 nL/min, as already reported (Chinello et al., 2015). Eluted peptides were ionized using nanoBoosterCaptiveSpray™ (Bruker Daltonics) source. MS acquisition was carried out using data-dependent-acquisition mode. CID fragmentation was promoted by N2 as collisional gas. Precursors of preferred charge state ranging from +2 to +5, with at least 1,575 counts were selected using a fixed cycle time of 5 s over the 300–2,000 Th window (excluding 1221.5–1,224 m/z) using IDAS (Intensity Dependent Acquisition Speed) and RT2 (RealTime Re-Think) options. To improve mass accuracy, in addition to a specific lock mass (1221.9906 m/z) a calibration segment was performed using 10 mM sodium formate cluster solution before beginning the gradient for each single run (Liu et al., 2015).

Exported MS2 data from DataAnalysisTM v.4.0 SP4 (Bruker Daltonics, Germany) were submitted to an in-house Mascot Server (Matrix Science, UK; v. 2.4.1), through Mascot Daemon. Database searching was restricted to human Swissprot (accessed Feb 2017, 553,655 sequences; 198,177,566 residues). Trypsin as enzyme and carbamidomethyl as fixed modifications were set in search parameters. One missed cleavage was allowed. Mass tolerances for all identified hits were set at 20 ppm for the precursor ions and 0.05 Da for the product ions. Automatic decoy database search was used to estimate the false discovery rate; peptide-to-spectrum matches were rescored through a built-in Percolator algorithm. Only proteins with at least one significant peptide with a score above 13 (*p* < 0.05) were considered. Progenesis QI for proteomics v.2.0.5556.29015 (Non-linear Dynamics, Newcastle, UK) was used as label-free quantification platform (Raimondo et al., 2015). Briefly, 3 runs for each samples (technical replicates) after DataAnalysisTM elaboration were imported as centroided data and were automatically aligned using a reference run to create a maximal overlay across the data. The peak picking was set using 0.2 min as minimal peak width, and a default sensitivity. Peptides were identified using an in-house Mascot search engine as described in the previous paragraph. Only non-conflicting peptides, not shared between different proteins, were used for the relative quantification.

### Bioinformatics analysis

The integrated network analysis was conducted with ProteinQuest™ platform^1^ and Panther (http://www.pantherdb.org/) on differentially expressed proteins (DEPs). Panther compares the proteins list uploaded with a reference proteome data set (human proteome), in order to identify the biological process over/under-represented in the uploaded list (Table S5). It assigns a *p*-value (applying Bonferroni correction for multiple testing) to each biological process based on its over/under-representation in the uploaded list. ProteinQuest™ highlights any relationship between several type of concepts and biological items using literature resources and plots the found relationships as networks between the chosen items. ProteinQuest™ output has been further checked to be sure that the shown relationships were based on experimental findings and not only due to the citation of two proteins in the same article. All plots for the explorative analysis of abundances, heatmap and clustering have been realized with the open source software R and Bioconductor repository, using ggplot2 and Heatplus packages (https://cran.r-project.org/; https://www.bioconductor.org/).

### Western blot

Seventy micrograms of the biopsies extracts were subsequently loaded on a polyacrylamide gel. The proteins were reduced with beta-mercaptoethanol and denaturated by boiling for 10 min in distilled water. The loaded 36 μL were composed of extracted proteins, RIPA lysis buffer and Laemmli 1X. After SDS-PAGE with a running buffer [running buffer 1X (Tris 25 mM, Glycine 200 mM and 0.1% SDS(Sodium dodecyl sulfate)] and a current of 7–80 Volt for the stacking step and 100 V for separating step, blotting was performed on 0.2 μm nitrocellulose membranes (Hybond-P, Amersham) over night at 200 mA. The membranes were blocked in non-fat dry milk (2 and 5%) in TBS buffer (Sigma) containing 0.1% Tween-20 at room temperature for 1 h with agitation.

Primary antibodies were incubated overnight in non-fat dry milk (2 and 5%) in TBS buffer containing 0.1% Tween-20. The following antibodies were used: anti-TYSY (clone TS106, Millipore, 1:250 dilution) in 2% non-fat milk and with 2% milk blocked; anti-DRY (sc-74593, Santa Cruz, 1:250 dilution) in 2% non-fat milk and with 2% milk blocked; anti-PUR2 (12031-S1, Abnova, 1:500 dilution) in 5% non-fat milk and with 5% milk blocked. After washing, membranes were incubated with a horseradish peroxidase-conjugated with a sheep anti-mouse secondary antibody (Amersham Biosciences, 1:5,000 dilution) for 45 min at room temperature in agitation. Membranes remained in agitation for about 2 h with TBS buffer to wash unbound antibodies away. The chemiluminescent detection system (ECL Plus, Amersham) has been spotted above the membranes and has been left for 5 min in the dark at room temperature before the development. The signal was detected on a X-ray film (Amersham). Different exposure times were adopted depending on the protein, ranging from a few seconds to few hours. The semi-quantitative results of protein expression on OC tissue lysates before therapy with PMX are reported as histograms.

For protein concentration normalization purposes, the membranes were colored with the nonspecific protein dye Ponceau S. We selected a section of the blot that covered a wide range of molecular weights in each case, typically approximately 90% or more of the lane length for each protein (Romero-Calvo et al., 2010). One-way ANOVA analysis was performed with the open source software R using stats package.

## Results

### Translational study

In our studies we identified ovarian cancer tissue samples that were collected from patients who received platinum drugs as first line therapy, showed drug resistance and were treated with PMX as second line therapy. We examined 52 frozen biopsies (received from the TOC bank) from 24 patients. All samples were histologically examined and only three samples of high-grade serous carcinoma collected before PMX treatment were considered suitable for proteomic studies to compare proteomes from patients who responded differently to PMX. The samples information are reported in Table S1 in the Supplementary Information.

### Protein set of reference design

**Figure 2:**
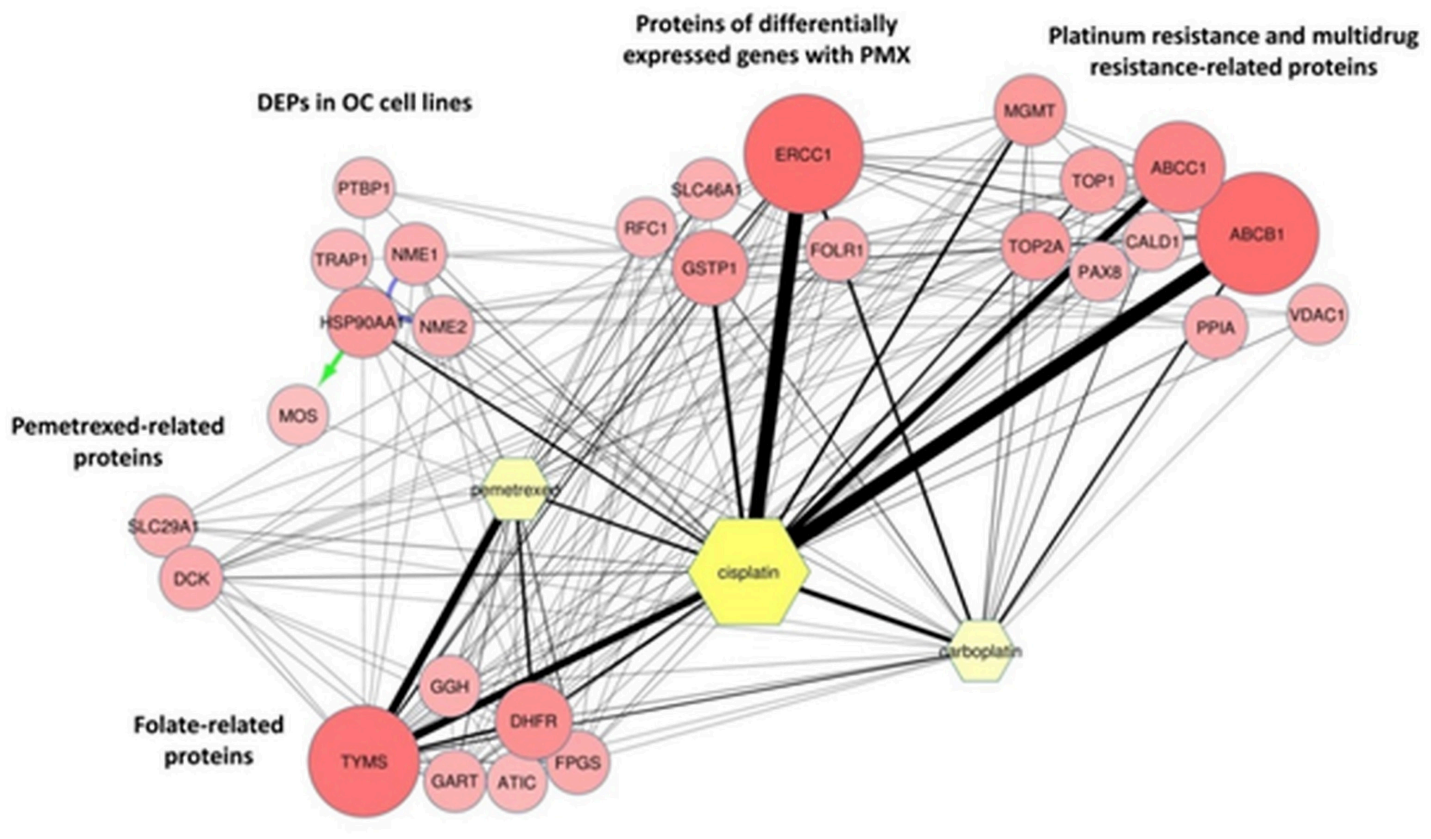
ProteinQuest™ network of the proteins belonging to the PSR. The yellow nodes show the drugs used for the search (PMX, cisplatin, and carboplatin) and the red circles show the proteins used as input. The lines indicate the relationships among the adjacent nodes; the thickness is proportional to the strength of the relationship. The figure shows both relationship based on biological database information (green and blue arrows) and relationship based on literature information (black connections) connections. The proteins were grouped as described in Table 1.

The MS proteomic analysis and selection of the proteins significantly modulated in the samples is usually performed through statistical metrics and validated after the final protein panel compilation. In our analysis, we adopted a Protein Set of Reference (PSR) as internal validation system. The PSR was designed by including proteins previously validated in cell-based experiments and others reported in the literature as relevant to PMX activity (metadata) (Figures 1A,B and Figure 2) (Taddia, 2015). Identification of a protein subset that characterizes the activity of folate metabolism targeted Ph.D. thesis. University of Modena and Reggio Emilia (unpublished data). The PSR was compiled before starting the MS study and has been used as an internal validation protein set that was compared with the actual protein profile obtained during the MS proteomic data elaboration and was used to define the final panel (Figure 1B). The PSR includes 28 proteins (Table 1, Figure 2) classified into 2 different groups (Group 1 and Group 2ì-2iv).

**Table 1:** Description of PSR and selection criteria.

**Selection criteria**	**UniProt Entry name**	**Protein name**	**Gene**
Group 1: Differentially expressed proteins (DEPs) from studies on OC cell lines (A2780) treated with PMX	HS90A_HUMAN	Heat shock protein HSP90-alpha	HSP90AA1
	TRAP1_HUMAN	Heat shock protein 75 kDa, mitochondrial; tumor necrosis factor (TNF) receptor-associated protein 1	TRAP1
	MOS_HUMAN	Proto-oncogene serine/threonine-protein kinase mos	MOS
	PTBP1_HUMAN	Polypyrimidine tract-binding protein 1	PTBP1
	NDKB_HUMAN	Nucleoside diphosphate kinase B	NME2
	NDKA_HUMAN	Nucleoside diphosphate kinase A	NME1
Group 2.i: Folate related proteins and proteins target of PMX	TYSY_HUMAN	Thymidylate synthase	TYMS
	DYR_HUMAN	Dihydrofolate reductase	DHFR
	FOLC_HUMAN	dihydrofolate synthase/Folylpolyglutamate synthase, mitochondrial	FPGS
	GGH_HUMAN	Gamma-glutamyl hydrolase	GGH
	PUR2_HUMAN	Trifunctional purine biosynthetic protein adenosine-3; glycinamide ribonucleotide formyltransferase	GART
	PUR9_HUMAN	Bifunctional purine biosynthesis protein PURH; 5-aminoimidazole-4-carboxamide ribonucleotide formyltransferase/IMP cyclohydrolase	ATIC
Group 2.ii: Other proteins connected to PMX	DCK_HUMAN	Deoxycytidine kinase\	DCK
	S29A1_HUMAN	Equilibrative nucleoside transporter 1	SLC29A1
Group 2.iii: Proteins derived from gene analysis studies on the response to PMX in platinum-resistant OC patients	ERCC1_HUMAN	DNA excision repair protein ERCC-1; excision repair cross-complementation group 1	ERCC1
	FOLR1_HUMAN	Folate receptor 1, alpha	FOLR1
	RFC1_HUMAN	Replication factor C subunit 1	RFC1
	GSTP1_HUMAN	Glutathione S-transferase Pi 1	GSTP1
	PCFT_HUMAN	Proton-coupled folate transporter	SLC46A1
Group 2.iv: Proteins associated with platinum and multidrug resistance	MRP1_HUMAN	Multidrug resistance-associated protein 1	ABCC1
	MDR1_HUMAN	Multidrug resistance protein 1	ABCB1
	TOP2A_HUMAN	DNA topoisomerase II-alpha	TOP2A
	TOP1_HUMAN	DNA topoisomerase I	TOP1
	CALD1_HUMAN	Caldesmon 1	CALD1
	PAX8_HUMAN	Paired box protein Pax-8	PAX8
	MGMT_HUMAN	Methylated-DNA–protein-cysteine methyltransferase	MGMT
	VDAC1_HUMAN	Voltage-dependent anion-selective channel protein 1	VDAC1
	PPIA_HUMAN	Peptidyl-prolyl cis-trans isomerase A	PPIA

First, we included proteins whose expression was modulated in previous proteomic studies of platinum-resistant cancer cell lines (A2780/CP) treated with PMX (Taddia, 2015). Identification of a protein subset that characterizes the activity of folate metabolism targeted Ph.D. thesis. University of Modena and Reggio Emilia (unpublished data) (Table 1: Group 1), namely, heat-shock protein 90A (HS90A), tumor necrosis factor (TNF) receptor-associated protein 1 (TRAP1), a proto-oncogene and serine/threonine kinase (MOS), polypyrimidine tract-binding protein 1 (PTBP1), nucleotide diphosphate kinase B (NDKB), and nucleotide diphosphate kinase A (NDKA) (left side, Figure 2). These proteins have been reported to be involved in the drug response of OC (Landriscina et al., 2010; Stecklein et al., 2012; Chu et al., 2013; Genovese et al., 2014).

Other members of the panel were retrieved from a literature-based analysis performed with ProteinQuest™ (http://www.proteinquest.com) (Table 1: Group 2). In particular, we searched for proteins connected to OC, PMX and platinum drugs (carboplatin and cisplatin), and gave this as input information for protein search. These proteins are: (2i) folate-related proteins and protein targets of PMX: TYSY, dihydrofolate synthase (DYR, DHFR), folylpolyglutamate synthase (FOLC), gamma-glutamyl hydrolase (GGH), PUR2, and 5-aminoimidazole-4-carboxamide ribonucleotide formyltransferase/IMP cyclohydrolase (PUR9) (2, 12); (2ii) other proteins connected to PMX: deoxycytidine kinase (DCK) and the equilibrative nucleotide transporter (S29A1) (Arik and Kulaçoglu, 2011; He et al., 2011; Li et al., 2012); (2iii) proteins from studies analyzing the response of genes to PMX in patients with platinum-resistant OC: excision repair cross-complementation group 1 (ERCC1), folate receptor 1 (FOLR1), replication factor C subunit 1 (RFC1), glutathione S-transferase Pi 1 (GSTP1), and proton-coupled folate transporter (PCFT) (Buqué et al., 2013); and (2iv) proteins associated with platinum and multidrug resistance: multidrug resistance-associated protein 1 (MRP1), multidrug resistance 1 (MDR1), topoisomerase II alpha (TOP2A), DNA topoisomerase I (TOP1), caldesmon 1 (CALD1), paired box protein 8 (PAX8), Methylated-DNA–protein-cysteine methyltransferase (MGMT), voltage-dependent anion selective channel 1 protein (VDAC1), and Peptidyl-prolyl cis-trans isomerase A (PPIA) (Dingemans et al., 2001; Hegi et al., 2005; Zhang et al., 2008; Auner et al., 2010; Ferrandina et al., 2010; De Pas et al., 2011; Indran et al., 2011; Maráz et al., 2011; Bar et al., 2012; Bock et al., 2012; Curtin, 2012; Melguizo et al., 2012; Mhawech-Fauceglia et al., 2012; Tyleckova et al., 2012; Johnatty et al., 2013; O'Shannessy et al., 2013; Kucukgoz Gulec et al., 2014).

### Label-free MS proteomic approach for OC biopsies

We applied the label-free proteomic approach to identify DEPs, in the selected biopsies. The MS analysis was performed with an online pLC coupled Impact HD™ mass spectrometer (Bruker Daltonics, Germany) and the results were processed using a robust bioinformatics analysis to identify the connections between protein profiles and the different response to PMX treatment.

Of the 2,863 identified proteins, 1,198 different protein IDs were DEPs in the two responders (complete, CR and partial, PR) compared with the non-responder one (NR) (Tables S2, S6, S7). The following filters were applied to elaborate the MS data using Progenesis QI for proteomics using as selection criteria a fold change ≥1.5 and ANOVA test *p* ≤ 0.05 and power ≥0.8.

**Figure 3:**
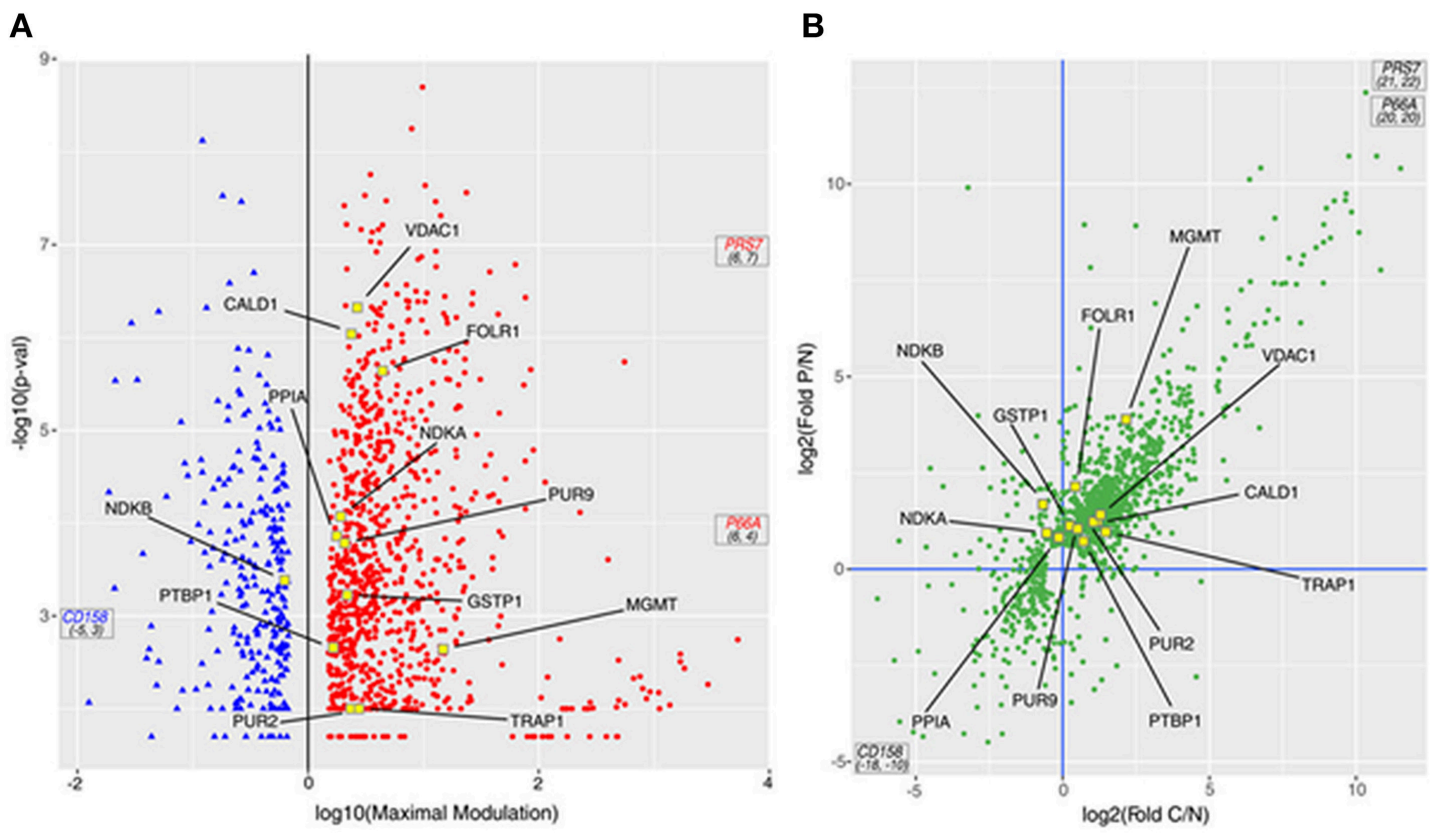
DEPs from the MS study. **(A)** Volcano plot of the 1,198 proteins; the *p*-value derived from an ANOVA of the elaborated MS data is reported as –log10 of the *p*-value vs. the maximal expression difference of the protein (Maximal Modulation) reported as a log10 value. The maximal expression difference is defined as the lower of the values between the CR/NR and PR/NR abundance ratios (C/N fold and P/N fold, respectively, in the graphs) for lower expressed proteins and the higher of the values between the CR/NR and PR/NR ratios (C/N fold and P/N fold, respectively, in the graphs) for higher expressed proteins. The blue and red spots symbolize the lower and higher expressed proteins, respectively. **(B)** Scatter plot of 1,198 proteins presented as the log2 value of the P/N fold (ratio between the MS abundance observed for PR and NR patients) vs. the log2 value of the C/N fold (ratio between the MS abundance observed for CR and NR patients). In both panels: (i) PRS7, P66A and CD158 proteins are out of scale due to their very large/small C/N and P/N ratio; therefore, they are marked with their coordinates. (ii) Proteins included in the PSR are indicated with yellow-outlined squares.

In Figure 3A a volcano plot shows the 1,198 DEPs in the responders compared with the NR patient. The *p-*value is presented on the Y-axis as –log10 (*p*-value) and ranges from 1.70 to 8.70 with respect to the maximal expression difference, which is presented on the X-axis as log10 (Maximal Modulation) and ranges from −5.45 to −0.17 for the lower expressed proteins (proteins less expressed in PR/CR than in NR; blue spots, on the left) and from 0.18 to 6.48 for the higher expressed proteins (proteins more expressed in PR/CR than in NR; red spots, on the right). The maximal expression difference is defined as the lower of the values between the CR/NR and PR/NR abundance ratios for lower expressed proteins and the higher of the values between the CR/NR and PR/NR ratios for higher expressed proteins. The proteins in the high –log10 (*p*-value) range show the lowest *p*-values and therefore represent the statistically most reliable proteins. Three proteins, coiled-coil domain-containing protein 158 (CD158), proteasome 26S protease regulatory subunit 7 (PRS7) and transcriptional repressor p66-alpha (P66A), were highly differentially expressed and show a maximal expression difference that exceeds the range examined and a *p*-value in the average range.

In particular, 698 proteins (Figure 3B, top-right quadrant) were higher expressed in both responders (CR and PR) compared with the NR patient; 220 proteins were higher expressed in one of the responders (CR or PR) compared with the NR patient, but not the other responder. Eight proteins (Figure 3B, bottom-right quadrant) were higher expressed in the CR patient compared with the NR patient and were lower expressed in the PR patient compared with the NR patient. Collectively, all 926 proteins were considered higher expressed proteins. One hundred thirteen proteins (Figure 3B, bottom-left quadrant) were lower expressed in both responders (CR and PR) compared with the NR patient; 120 proteins were lower expressed in one of the responders (CR or PR) compared with the NR patient, but not the other responder. Thirty-nine proteins (Figure 3B, top-left quadrant) were lower expressed in the CR patient compared with the NR patient, but were higher expressed in the PR patient compared with the NR patient. Collectively, all 272 proteins were considered lower expressed proteins.

Twelve out of 28 proteins in the PSR (Table 1, yellow dots in Figure 3) were detected and quantified by the MS analysis. Among the 12 proteins, six (TRAP1, PTBP1, PUR2, CALD1, MGMT, and VDAC1) were higher expressed in both responders (CR and PR; Figure 3B, top-right quadrant); four proteins (PPIA, GSTP1, FOLR1, and PUR9) were higher expressed in the PR patient but were not significantly modulated in the CR patient (Figure 3B, top quadrants). One protein, NDKB, was lower expressed in the CR patient and higher expressed in the PR patient (Figure 3B, top-left quadrant). NDKA was higher expressed in the PR patient, whereas its level was not significantly altered in the CR patient (Figure 3B, top-left quadrant).

### Biological process analysis and selection

The 1,198 DEPs were processed with PANTHER and ProteinQuest™ using their annotation to highlight any common biological processes in which they are involved and to identify their interconnections. The UniProt Entry Names were used for the statistical over-representation test in PANTHER (http://pantherdb.org version 10.0, reference list: Homo sapiens). Eight hundred forty-nine proteins were classified into 135 biological processes, 19 of which were statistically significant because of very relevant or not relevant for the PMX effect (namely over or under-represented) with a *p* < 0.05 (Table S3).

**Figure 4:**
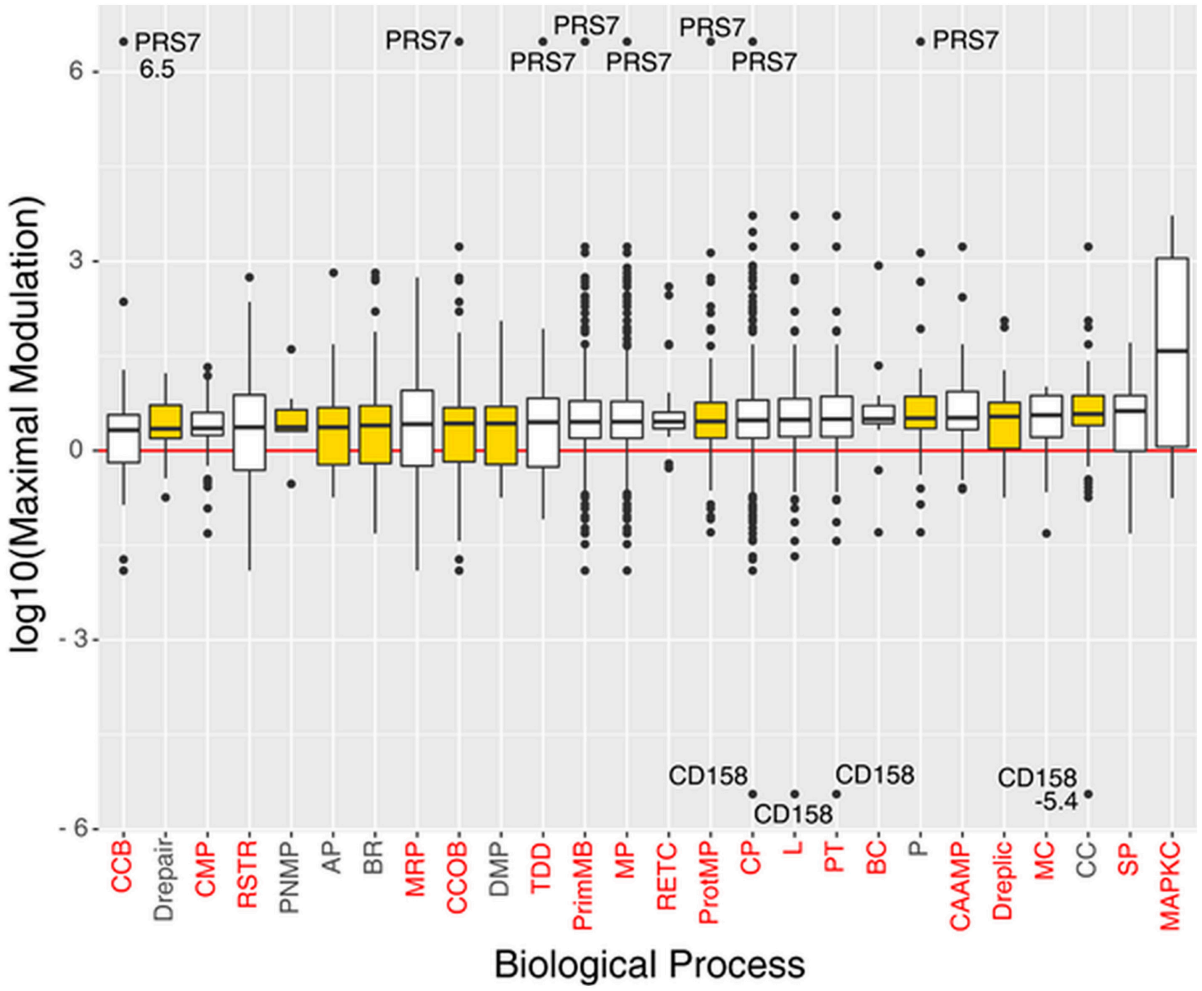
Boxplots of the proteins log10(maximal modulation) (maximal expression difference) values (y-axis), associated to significant and/or selected (yellow box) biological processes (x-axis), sorted by median value. Each biological process is indicated with its acronym (see Table S3). Biological processes that have a *p* < 0.05 in the Panther's statistical over-representation test are written in red. Proteins PRS7 and CD158 were labeled with their maximal modulation value due to their very high expression difference.

The 135 biological processes are listed in the Supplementary Information (SI; Table S4); we filtered the list of the biological processes down to 10 members according to their relevance to OC, evaluated on the basis of literature information (metadata) and on the basis of the relevance of the processes associated to PMX drug treatment that contain proteins belonging to the PSR (Table 1). Four hundred sixty proteins belonging to the selected processes were considered for further bioinformatic analysis. We observed that 8 of 12 proteins of the PSR were also experimentally detected using MS in our experiments; in fact they were found among the 460 proteins mentioned above (Table 1). Figure 4 shows a box plot representation of the maximal expression difference values of the proteins that belong to each of the 19 statistically significantly over/under-represented processes, as well as the other selected processes; the 10 selected processes are plotted in yellow.

In particular, we focused our attention on processes involved in cellular component organization or biogenesis, protein metabolism, proteolysis, DNA repair, DNA replication, DNA metabolism, purine nucleobase metabolism, biological regulation, the cell cycle, and apoptosis.

The biological processes we selected are mainly related to neoplastic disease (Table 2). The general cancer-related processes include the genetic control of cell replication (CC) resulting in massive cell proliferation, and apoptosis (AP), which is essential for maintaining the physiological balance between cell death and cell survival. Cellular component organization (CCOB) includes a number of transcription factors, growth factors and receptors. Specifically, in epithelial cells such as ovarian epithelial cells, a distinctive apical-basal polarity is the physiological morphology, whereas a loss of polarity is frequently assumed to be a common symptom of cancer progression. Moreover, the typical neoplastic behavior includes aberrant tissue and cellular organization and signal transduction. The purine nucleobase metabolic process (PNMB) involves folate-related proteins and key proteins associated with the PMX mechanism of action and proteins required for DNA replication (Dreplic) in cells, such as cancer cells. The neoplastic process and its regulation (BR, Drepair) are characterized by mutations in oncogenes and tumor suppressor genes that cause alterations in multiple intracellular signaling pathways and affect tumor cell metabolism (DMP), re-engineering the tumor cell metabolism to allow enhanced survival and growth. Finally, proteases (P, ProtMP) are important in multiple processes during malignant progression, including tumor angiogenesis, invasion and metastasis. Thus, proteolysis networks that involve different constituents of the tumor microenvironment and key proteases have been chosen as important biological processes for future evaluations.

**Table 2:** Selected biological processes description using PANTHER GO-Slim, and number of proteins.

**Biological process ID**	**PANTHER GO-slim biological process**	**N° proteins**
CCOB	cellular component organization or biogenesis	181
ProtMP	protein metabolic process	200
Dreplic	DNA replication	23
PNMB	purine nucleobase metabolic process	13
BR	biological regulation	97
P	Proteolysis	54
DMP	DNA metabolic process	35
CC	cell cycle	69
AP	apoptotic process	27
Drepair	DNA repair	11

The set of 460 proteins was then enriched with some proteins considered relevant to our study that were not included by PANTHER during the selection process; these proteins are part of the PSR. In particular PTBP1 plays a role in pre-mRNA splicing and in regulating alternative splicing events. VDAC1 forms a channel through the mitochondrial outer membrane and the plasma membrane. It is involved in regulating cell volume and apoptosis. FOLR1 binds folic acid and reduced folates and mediates the transport of 5-methyltetrahydrofolate and folate analogs into cells. Moreover, FOLR1 is involved in folic acid metabolism and is connected to purine nucleobase metabolism. It is also a known marker of OC, as it is overexpressed in this cancer type (Kalli et al., 2008). Finally, GSTP1 plays an important role in detoxification by catalyzing the conjugation of many hydrophobic and electrophilic compounds into several metabolic processes.

### Protein network analysis and final panel definition

**Figure 5:**
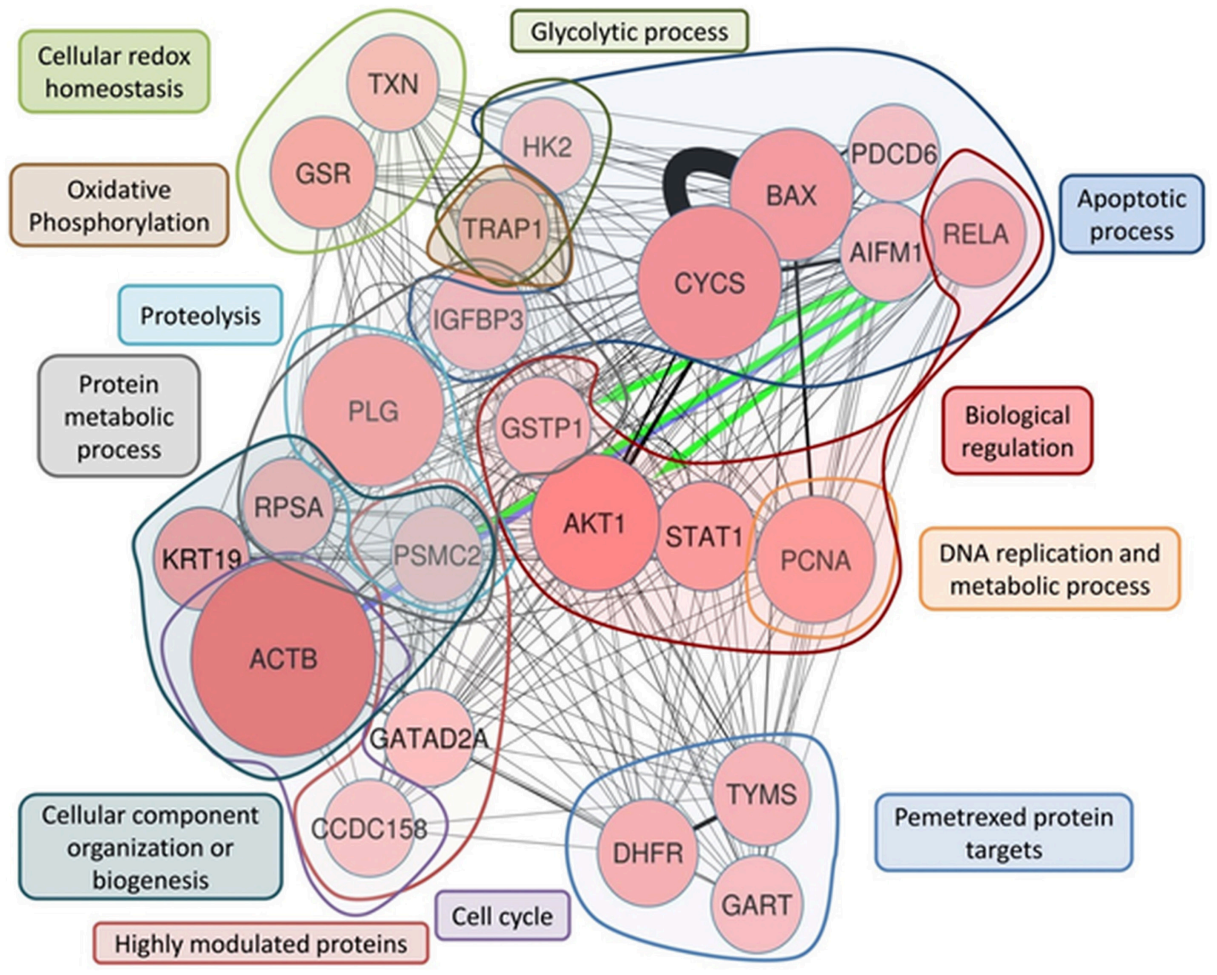
ProteinQuest™ network of the final proteins panel. The red circles show the proteins used as the input. The lines indicate the relationships among adjacent nodes; the thickness is proportional to the strength of the relationship. The figure shows both relationships based on biological database information (green and blue arrows) and relationship based on literature information (black connections) connections Proteins were grouped based on the biological process in which they are involved. The blue islet represents the proteins involved in the apoptotic process, the red islet indicates proteins implicated in biological regulation, and the orange islet shows proteins related to DNA replication and metabolic processes. The violet islet represents cell cycle proteins, the dark blue islet indicates proteins involved in the cellular component organization or biogenesis biological process, and the gray islet shows the proteins involved in metabolic processes. The proteins involved in proteolysis are highlighted in light blue, and the proteins shown in brown are involved in oxidative phosphorylation. The light-green islet represents proteins related to cellular redox homeostasis and the dark green islet indicates proteins involved in the glycolytic biological process. Additionally, TYSY (TYMS), DYR (DHFR), and PUR2 (GART), which are PMX protein targets, are highlighted in the light blue islet, and the rose islet shows the highly different expressed proteins (see Table 3).

**Figure 6:**
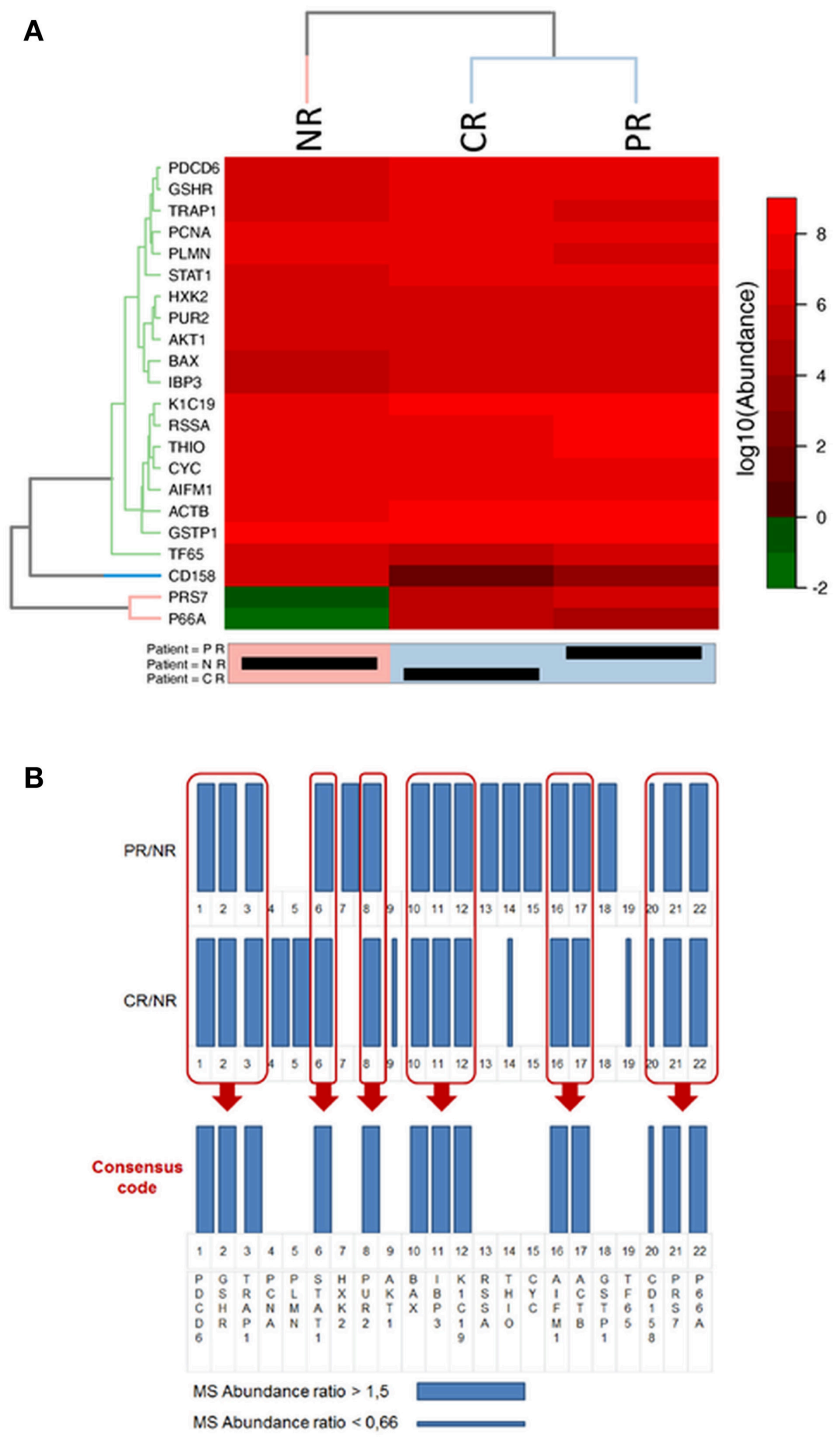
Representations of the final selected protein panel obtained from the MS analysis. **(A)** Heat-map of the logarithmic values of the MS abundances of 22 proteins in the final panel. The reported dendrogram was built based on the dissimilarity matrix using Euclidean distances and the average linkage method. For the dendrogram, a cut-off of 3 was used for the proteins, and a cut-off of 6 was used for the patients. NR, non-responder; CR, complete responder; PR, partial responder. In the heatmap representation the abundance values is in logarithmic scale in base 10, thus differences between CR, PR, and NR are clearly visible only if a difference of at least one order of magnitude in the protein abundance values exists. **(B)** Bar code representation of the MS abundance ratios (CR/NR and PR/NR) of the 22 proteins in the final panel. Thick bars represent higher expressed proteins, whereas thin bars represent lower expressed proteins. The bar code representation of the ratios of the abundance values (CR/NR and PR/NR); thus differences between CR, PR, and NR are clearly visible when the ratio are higher than 1.5 or lower than 0.66.

The ProteinQuest™ platform was queried using the 464 proteins selected through the biological process analysis as input. The set of proteins was filtered based on their associations with OC, cisplatin or carboplatin, and drug resistance; these terms were used as keywords for protein selection. A panel of 18 proteins was obtained from the selection process (Table 3). The applied filters are related to the disease and the patient's therapeutic features. We decided to integrate the panel of the 18 proteins with some other relevant proteins that include the main biological targets of PMX, TYSY, DRY, and PUR2, and 3 DEPs, namely CD158, P66A, PRS7. Among the three target proteins, thymidylate synthase (TYSY) and dihydrofolate reductase, DRY, were not detected through MS experiments because of their low levels, below the limit of detection, while glycinamide ribonucleoside transferase (PUR2) was detected in the MS experiments but excluded from the selection; the 3 DEPs showed the greatest change in expression obtained from the MS experiments (CD158, P66A, PRS7) (Table 3, Figures 5, 6) and therefore we believed that they are important. The most relevant biological processes, including the selected panel, were related to biological regulation, apoptosis, cellular component organization or biogenesis, protein metabolism, DNA metabolism, DNA replication, proteolysis, purine nucleobase metabolism, the cell cycle, cell redox homeostasis, the glycolytic process and oxidative phosphorylation (Table 3, Figure 5).

**Table 3:** Final panel of 24 proteins: function and involvement in cancer mechanisms.

**UniProt entry name**	**Protein name**	**Gene**	**PANTHER GO-slim biological process**	CR/NR^*^	PR/NR^*^
AKT1_HUMAN	RAC-alpha serine/threonine-protein kinase	AKT1	biological regulation	0.52	0.93
BAX_HUMAN	Apoptosis regulator BAX	BAX	apoptotic process biological regulation cellular component organization or biogenesis	3.90	4.76
CYC_HUMAN	Cytochrome c	CYCS	apoptotic process biological regulation	1.29	1.91
GSHR_HUMAN	Glutathione reductase, mitochondrial	GSR	protein metabolic process	1.58	2.52
GSTP1_HUMAN	Glutathione S-transferase Pi 1	GSTP1	*PantherDB: unclassified*	1.19	2,.18
HXK2_HUMAN	Hexokinase-2	HK2	biological regulation	1.17	2.04
IBP3_HUMAN	Insulin-like growth factor-binding protein 3	IGFBP3	biological regulation	1.58	1.63
K1C19_HUMAN	Keratin, type I cytoskeletal 19	KRT19	cellular component organization or biogenesis	2.27	2.36
PCNA_HUMAN	Proliferating cell nuclear antigen	PCNA	biological regulation DNA metabolic process DNA replication	1.81	1.36
PDCD6_HUMAN	Programmed cell death protein 6	PDCD6	proteolysis protein metabolic process	207	2.88
RSSA_HUMAN	40S ribosomal protein SA	RPSA	cellular component organization or biogenesis protein metabolic process	0.98	1.65
STAT1_HUMAN	Signal transducer and activator of transcription 1-alpha/beta	STAT1	biological regulation	19.29	11.03
TRAP1_HUMAN	Heat shock protein 75 kDa, mitochondrial; tumor necrosis factor (TNF) receptor-associated protein 1	TRAP1	protein metabolic process	2.77	1.95
THIO_HUMAN	Thioredoxin	TXN	biological regulation protein metabolic process	0.59	2.68
ACTB_HUMAN	Actin, cytoplasmic 1	ACTB	cell cycle cellular component organization or biogenesis	12.74	26.32
AIFM1_HUMAN	Apoptosis-inducing factor 1, mitochondrial	AIFM1	protein metabolic process	4.20	2.38
PLMN_HUMAN	Plasminogen	PLG	proteolysis protein metabolic process	2.33	0.82
TF65_HUMAN	Transcription factor p65	RELA	biological regulation	0.11	1.35
TYSY_HUMAN	Thymidylate synthase	TYMS	*PantherDB: unclassified*	—	—
DRY_HUMAN	Dihydrofolate reductase	DHFR	*PantherDB: unclassified*	—	—
PUR2_HUMAN	Trifunctional purine biosynthetic protein adenosine-3; glycinamide ribonucleotide formyltransferase	GART	purine nucleobase metabolic process	2.07	2.34
CD158_HUMAN	Coiled-coil domain-containing protein 158	CCDC158	cell cycle	<3.6 × 10^−6^	>1.1 × 10^−3^
P66A_HUMAN	Transcriptional repressor p66-alpha	GATAD2A	*PantherDB: unclassified*	>1.4 × 10^6^	>0.7 × 10^6^
PRS7_HUMAN	Proteasome 26S protease regulatory subunit 7	PSMC2	cellular component organization or biogenesis proteolysis protein metabolic process	>1.5 × 10^6^	>3.0 × 10^6^

* *Abundance ratio; CR, complete responder; PR, partial responder; NR, non-responder*.

The panel of 24 proteins is presented in Table 3 along with the main recognized roles of the proteins and the processes in which they are involved.

To highlight the metabolic connections of the selected 24 protein panel, we interrogated ProteinQuest™ with the proteins as input data and obtained the network reported in Figure 5.

Figure 5 illustrates the network representing the metabolic connection of the 24 selected proteins, which provide a functional picture (biological processes) of the integration of the selected protein features, thereby proposing a predictive model for drug responses. To rationally address the functional role of the 24 panel network we thoroughly examined the functions of the selected proteins and their involvement in cancer mechanisms and drug responses using literature information.

With the aim of understanding if the selected proteins were significantly differently expressed in CR and PR with respect to NR, we used the heat map clustering method.

Figure 6A shows the heat-map of the logarithmic values of the MS abundances of 22 proteins in the final panel. The patient clustering (Euclidean distance, dendrogram cut = 6.0, complete linkage clustering) shows a similarity between the CR and PR patients compared with the NR patient. In Figure 6B, the MS abundance ratios (CR/NR and PR/NR) for the 22 proteins in the final panel are represented as bar codes. Thirteen of 22 proteins form a consensus bar code protein change that characterizes the responders: PDCD6, GSHR, TRAP1, STAT1, PUR2, BAX, IBP3, K1C19, AIFM1, ACTB, CD158, PRS7, and P66A.

From the heat-map clustering analysis the CR and PR show a different protein expression with respect to NR thus supporting the concept that the 24 protein panel is predictive for PMX response in the present study.

### Western blot studies

**Figure 7:**
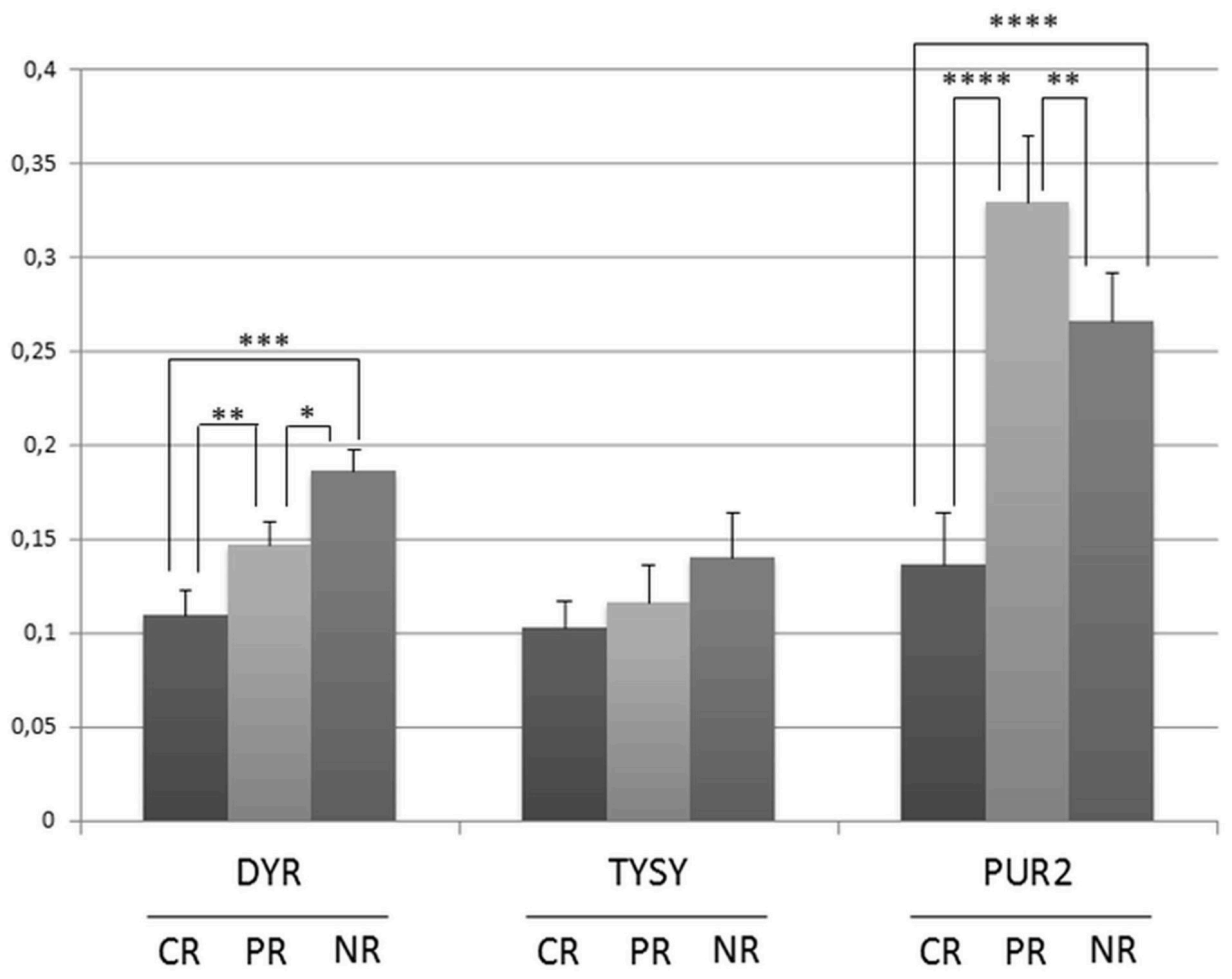
Semi-quantitative analysis of proteins expression in OC tissue lysates before therapy with PMX. Histograms represent the densitometric values for each protein normalized to the respective Ponceau S-stained gel. Values are means±s.e. ^*^ *p* < 0.05, ^**^p < 0.01, ^***^p < 0.001, ^****^p < 0.0001 by ANOVA test.

Lysates were obtained from the three selected biopsies and were subjected to a western blot (WB) analysis of the known PMX protein targets (TYSY, DYR, and PUR2) (Figure 7). The immunoblot technique allowed us to identify low-abundance proteins, such as proteins in the folate pathway, thus overcoming the concentration limits imposed by the MS analysis. In our case, both TYSY and DRY were not detected by MS, but literature data show that they are modulated by PMX. The CR patient showed lower expression of TYSY and DRY than the PR and NR patients; intermediate levels of these proteins were detected in the PR patient, and higher levels were observed in the NR patient. These results are consistent with the results reported by Huang. et al., who showed that the expression of the TYSY protein is associated with the cancer prognosis (Huang et al., 2015). PUR2 is the only protein that was related to PMX activity and detected with MS. Therefore, we performed the WB assay to validate the MS finding. PUR2 expression varies to an extent similar to the results obtained in the MS experiments.

## Discussion

In platinum-resistant OC tissues, a protein profile associated with the PMX response was related to a consistent set of proteins using a combined bioinformatics/MS proteomic approach. This panel includes proteins related to the PMX mechanism of action and was examined using an MS proteomic analysis of tissue samples from the patients. Due to difficulties in finding tissue samples with the wanted characteristics we could only select three patients who received chemotherapy and platinum drug and were treated further with PMX in second or third line therapy. The high heterogeneity of the tissue sample set allowed the selection of three pre-treatment tissue samples. The proteomic analyses highlight a panel of proteins whose levels were significantly different in samples obtained from patients before they received treatment. The global interpretation of the MS data was performed through bioinformatics analysis and was assisted by a PSR designed *ad hoc*.

We identified 24 proteins (Table 3) that might be used as a protein signature to guide the choice of treatment with PMX when standard first-line treatments have failed. The limitation of this study is the availability of only one sample for each patient category (CR, PR, NR), therefore it can be considered as a proof of concept study. Future evaluations will be directed toward the retrospective validation of the panel in a higher number of patient's samples.

Each of the identified proteins is also studied in several cancers other than OC. However, the set of 24 proteins and their variation in patients CR, PR, and NR, can be considered as a protein signature specific for the OC sub-population of this study, as reported in the bar code frame in Figure 6. The heat-map of the 24 proteins provides the quantitative outcomes that characterize the NR patient compared with the PR and CR patients. The protein levels observed in the NR patient significantly differ from that in the PR and CR patients. WB analyses of PMX target proteins showed that TYSY and DYR levels differ in a manner consistent with the literature, whereas PUR2 varies in a manner consistent with the data from the MS experiments. Neither TYSY nor DYR were detected in the MS experiments, but their levels were higher in the NR with respect to the CR and PR, in the WB.

We have described the biological role of the proteins included in the final set, in a cancer disease status and found a rationale that may explain their modulation in MS experiments.

The insulin-like growth factor (IGF) binding protein 3 (IBP3) belongs to a family of six proteins that transport IGF-I and IGF-II in the blood circulation and regulate their access to the potentially oncogenic IGF-I receptor (IGF1R) (Baxter, 2014). Several studies describe an inhibitory effect of IBPs on IGF-dependent tumor growth and drug resistance. IBP3-mediated methylation is associated with disease progression and death in patients with OC, suggesting that a reduction in the IBP3 levels may be a useful prognostic marker of disease progression and death (Wiley et al., 2006). According to several basic and translational studies (Denduluri et al., 2015), IGF pathway modulators might have promising effects when used to treat various malignancies. The IGF signaling pathway appears to play an important role in the drug resistance of OC. In A2780 ovarian carcinoma cells, drug resistance to either cisplatin or the combination of cisplatin and Taxol is correlated with the upregulation of IGF-1R expression. Furthermore, primary tumors collected from patients after 3/4 cycles of carboplatin-Taxol treatment also exhibit increased IGF-1R expression (Singh et al., 2014).

The expression of IBP3, keratin, type 1 cytoskeletal 19 (K1C19), and other genes is decreased in platinum-resistant ovarian carcinoma cell lines that were derived from the parental line by exposing the cells to increasing concentrations of cisplatin. The altered expression of these proteins suggests that they are involved in the process of drug resistance (Macleod, 2005).

Thioredoxin reductases (THIO) and glutathione reductase (GSHR) are fundamental enzymes that participate in the defense against oxidative damage related to oxygen metabolism. The importance of the redox status in the drug resistance of many types of cancers is well known. Recent studies have reported a critical role for oxidative stress in the development of drug resistance in epithelial OC (EOC). Two OC cell lines (MDAH-2774 and SKOV-3) and their chemo-resistant counterparts have been compared and the results showed a significant decrease in the levels of the GSHR mRNA levels and GSHR activity in both cisplatin-resistant cell lines, suggesting that the development of drug resistance might be caused by a pro-oxidant state in these cells (Belotte et al., 2014). Although tumor cells have long been considered defective in mitochondrial respiration and primarily depend on glycolytic metabolism (the Warburg effect), recent observations provide contrasting evidence showing that melanoma cells and breast cancer cells are critically dependent on oxidative phosphorylation (OXPHOS) rather than glycolysis (Haq et al., 2013; LeBleu et al., 2014).

Notably, the most aggressive OC cell lines show a marked dependence on glutamine rather than glucose (Yang et al., 2014). In addition, cancer stem cells obtained from patients with EOC preferentially utilize OXPHOS and resist glucose deprivation (Pastò et al., 2014). Recently, TRAP1 has been shown to play important roles in regulating oxidative metabolism in OC cells. A strict correlation between low TRAP1 expression and oxidative metabolism in OC cells is responsible for the progression of cancer and the response to platinum-based therapies through the activation of an inflammatory program (Matassa et al., 2016). The same study revealed that the multidrug resistance proteins transporter 1, ATP binding cassette subfamily B member (TAP1) and MDR1, which belong to the MDR subfamily, are key mediators of metabolism-driven and inflammation-induced drug resistance. Hexokinase-2 (HK2), the first rate-limiting enzyme in the glycolytic pathway, is known to be up-regulated in many cancers, and its overexpression correlates with short progression-free survival, which may be associated with chemoresistance in EOC (Suh et al., 2014). HK2 inhibits mitochondrial apoptosis through direct insertion in the mitochondrial outer membrane (Mathupala et al., 2006) and by inhibiting cytochrome c (CYC) release (Pastorino et al., 2002).

Based on recent evidence, deregulation of the pro- and anti-apoptotic pathways is a key factor involved in the onset and maintenance of chemoresistance in OC (Fraser et al., 2003). Among the regulators of apoptosis, BAX (a member of the Bcl-2 gene family) strongly influences the response of OC to cisplatin-based chemotherapy. The BAX expression level displays a predictive value in patients with OC who are treated with platinum-based chemotherapy, and appears to be the strongest prognostic indicator of disease-free survival (DFS) in the TP53(+) patient group (Kupryjanczyk et al., 2003). The expression level of programmed cell death 6 (PDCD6), a recently discovered pro-apoptotic protein, is down-regulated in cancer cell lines and OC tissues compared with that in normal cells and tissues (Park et al., 2012). The results of this study highlight the key roles of PDCD6 and cisplatin in tumorigenesis. The ectopic expression of PDCD6 in the SKOV-3 OC cell line combined with cisplatin treatment is more effective in suppressing cell growth than the drug treatment alone. Furthermore, the authors provide evidence that PDCD6 mediates the pro-apoptotic activity of cisplatin by down-regulating the nuclear factor-κB (NF-κB) pathway.

The transcription factor p65 (TF65) is a REL-associated protein involved in NF-κB heterodimer formation, nuclear translocation and activation. As shown in a recent *in vitro* analysis of EOC cell lines, epidermal growth factor receptor (EGFR) activates NF-kB transcription, which in turn induces the secretion of interleukin-6 (IL-6) and plasminogen activator inhibitor (PAI-1). PAI-1 inhibits urokinase plasminogen activator (UPA), an enzyme responsible for the cleavage of plasminogen (PLMN) to produce plasmin. In the same study, the authors proposed that EGFR, NF-kB, IL-6, and PAI-1 may be novel prognostic markers and might have a significant impact on therapy for a particular subset of patients with EOC (Alberti et al., 2012).

The signaling cascade that involves phosphoinositide 3-kinase (PI3K), AKT, and mammalian target of rapamycin (mTOR) is the most frequently altered pathway in human cancer and controls many of the processes that play important roles in mediating cancer onset and development (cell survival, mobility, metabolism, the cell cycle, angiogenesis, genomic instability and chemoresistance) (Fruman and Rommel, 2014). OC often exhibits alterations in one or more components of this signaling pathway, including the mutation and/or amplification of the oncogenes AKT1 and AKT2 (Cheng et al., 1992; Carpten et al., 2007). Notably, a phase I trial has been conducted using a combination of perifosine, an AKT inhibitor, and docetaxel in patients with recurrent OC. The treatment was more effective when the PI3K/AKT pathway was activated (Fu et al., 2012). Although activation of the PI3K/AKT pathway appears to be required for a better response to treatment, Hanrahan et al. (Hanrahan et al., 2012) reported that this pathway is necessary, but not sufficient, to confer sensitivity to the AKT-selective inhibitor. Cells that contain RAS pathway alterations or a loss of retinoblastoma-associated protein (RB1) are resistant to AKT inhibition, regardless of whether the PI3K/AKT pathway is activated.

Furthermore, cisplatin-induced apoptosis proceeds, at least in part, via a caspase-independent mechanism that involves apoptosis-inducing factor 1 (AIFM1), and in OC, AKT activation confers resistance to cisplatin-induced apoptosis by blocking this pathway (Yang et al., 2008).

Cisplatin-resistant cells often exhibit decreased uptake of cisplatin in combination with decreased endocytosis. Two major carboplatin-binding proteins, filamin and beta-actin (ACTB), are implicated in this process. Decreased expression of these two proteins was observed in two different human cisplatin-resistant cell lines (KB-CP20 and 7404-CP20) compared with the expression in their parental cell lines (KB-3-1 and BEL-7404) (Dasari and Tchounwou, 2014). Furthermore, the ACTB protein was up-regulated in patients with advanced-stage OC who responded to carboplatin and paclitaxel chemotherapy compared with non-responders (Sehrawat et al., 2016).

Notably, the 40S ribosomal protein SA (RSSA) is up-regulated in A2780 cisplatin-resistant cell lines compared with the levels observed in the parental A2780 cell line. This ribosomal protein also functions as a cell surface receptor for laminin. Laminin has been implicated in a wide variety of biological processes, including cell adhesion, differentiation, migration, metastasis and drug resistance. Thus, the overexpression of RSSA, which has been observed in many cancers, suggests that it has potential roles in tumor progression and drug resistance (Al-Eisawi et al., 2016).

Finally, we identified other relevant proteins that are potentially involved in the drug resistance of OC, namely, proliferating cell nuclear antigen (PCNA), signal transducer and activator of transcription 1-alpha/beta (STAT1), and GSTP1. The mechanisms by which they exert their action are diverse, ranging from cell cycle control, DNA methylation, DNA replication and repair (PCNA) (Hartmann et al., 1992) to drug metabolism (GSTP1). GSTP1 has an important role in cisplatin and carboplatin metabolism in OC cells, and alterations in GSTP1 expression may therefore influence the response of patients with OC to chemotherapy (platinum-based) (Sawers et al., 2014). Interestingly, a significant increase in apoptosis after platinum treatment was observed following the knockdown of STAT1 in resistant OC cells (Stronach et al., 2011).

Three proteins that were highly deregulated in the CR patient compared with the NR patient are 26S protease regulatory subunit 7 (PRS7), transcriptional repressor p66-alpha (P66A) and coiled-coil domain-containing protein 158 (CD158). PRS7 is a component of the 26S proteasome that is involved in protein degradation and is required for the maintenance of protein homeostasis by removing misfolded or damaged proteins. Notably, this system has well-known roles in regulating the sensitivity of OC to treatment (Bazzaro et al., 2006). P66A is a structural component of the nucleosome remodeling and deacetylase (NuRD) complex, which regulates gene expression at the chromatin level. Based on emerging evidence, modifications in NuRD subunits modulate their function within the complex, potentially providing additional drug targets (Lai and Wade, 2011).

Overall the present discovery proteomic study shows a behavior that is consistent with the response to PMX treatment.

We previously performed a study on cancer cells using an investigational peptide candidate to evaluate the protein signature associated with its mechanism of action. In those experiments, we used a knowledge-based approach combined with MS proteomics and bioinformatics. In the present work, we have transferred a similar approach to the study of tissue samples with the aim of identifying a protein set by implementing an alternative experimental design. The two studies show different applications of the proteomic studies that encompass the experimental design, sample management and global data interpretation. However, they both demonstrate how the combination of different approaches with a metadata-based protein selection drive to the final protein set.

The relevant methodological aspect of the present study is the design of a PSR as a tool for protein selection. The final panel identified represents a proof of concept that requires validation in a higher number of samples to evaluate its capacity to predict weather patients with OC who have received multiple treatments and are resistant to platinum drugs will respond to PMX.

## Author contributions

JS and EB performed the clinical study; LL performed the histopathological study; LS, LT, FG, and AG performed sample preparation for MS analysis and western blot; FM, CC, and MS performed the MS experiments and provided data elaborations tools; SFe and SFo performed the statistical analysis; LS, GG, and GM performed western blot analysis; DD, LL, CM, and AL performed the biological data analysis; SFe and MC conceived the work and wrote the manuscript.

### Conflict of interest statement

The authors declare that the research was conducted in the absence of any commercial or financial relationships that could be construed as a potential conflict of interest.

## Footnotes

^1^For information about ProteinQuest software, contact Prof. Enrico Bucci at the following address http://enrico.bucci@resis-srl.com.

## Supplementary material

The MS proteomics data have been deposited to the ProteomeXchange Consortium via the PRIDE (Vizcaíno et al., 2016) partner repository with the dataset identifier PXD007056. Mascot.dat file can be read with open-source software:

1. Mascot ^*^.dat files can be converted into pepXML e.g., with Mascot2XML in TPP.

2. Mascot results can be opened with the “MascotDatfile library” (see https://github.com/compomics/mascotdatfile).

Table S1 Clinical history and histological analysis information about the 3 samples studied in the present work and selected from the whole 52 samples set.

Table S2 Clinical history and histological analysis information about the 52 samples of the collection.

Table S3 DEPs from MS study. In “peptide” column, values in brackets refer to unique peptides.

Table S4 Biological processes analysis: significant and selected biological processes.

Table S5 List of Panther-DB biological processes analysis.

Table S6 List of peptide measurements from Progenesis QI.

Table S7 List of proteins measurements from Progenesis QI.
