# Supplementary material for: Proteomic and Bioinformatic Studies for the Characterization of Response to Pemetrexed in Platinum Drug Resistant Ovarian Cancer
Source: Front Pharmacol. 2018 May 8;9:454. doi: 10.3389/fphar.2018.00454 (PMC5952181; doi:10.3389/fphar.2018.00454)
Supplement: Table S1 — Clinical history and histological analysis information about the 3 samples studied in the present work and selected from the whole 52 samples set. [file Table_1.DOCX]

**Table S1.** Clinical history and histological analysis information about the 3 samples studied in the present work and selected from the whole 52 set.

| Patient | Age | Stage | First line treatment | Second line treatment | PMX treatment |
| --- | --- | --- | --- | --- | --- |
| CR | 48 | 2B | Taxol/Carboplatin  6 cycles | Combination containing platinum drugs  (3 cycles) | 4 cycles |
| PR | 63 | 3C | Taxol/Carboplatin  6 cycles | Other drugs  (doxorubicin liposomal pegylated)  8 cycles | 8 cycles |
| NR | 37 | 3B | Taxol/Carboplatin  6 cycles | Other drugs  (doxorubicin liposomal pegylated)  8 cycles | 4 cycles |
